# Indoor air quality and sick building syndrome symptoms in administrative office at public university

**DOI:** 10.1016/j.dialog.2024.100178

**Published:** 2024-04-12

**Authors:** Amalina Abu Mansor, Samsuri Abdullah, Aimi Nursyahirah Ahmad, Ali Najah Ahmed, Mohammad Fakhratul Ridwan Zulkifli, Suriani Mat Jusoh, Marzuki Ismail

**Affiliations:** a Institute of Tropical Biodiversity and Sustainable Development, Universiti Malaysia Terengganu, Kuala Nerus, 21030, Terengganu, Malaysia; b Faculty of Ocean Engineering Technology, Universiti Malaysia Terengganu, 20130, Kuala Nerus, Terengganu, Malaysia; c School of Engineering and Technology, Sunway University, Bandar Sunway, Petaling Jaya 47500, Malaysia; d Faculty of Science and Marine Environment, Universiti Malaysia Terengganu, 20130, Kuala Nerus, Terengganu, Malaysia

**Keywords:** Indoor air quality, Sick building syndrome symptoms, Relative humidity, Headaches

## Abstract

Sick Building Syndrome (SBS) is an illness among workers linked to time spent in a building. This study aimed to investigate the Indoor Air Quality (IAQ) and symptoms of Sick Building Syndrome (SBS) among administrative office workers. The IAQ parameters consist of ventilation performance indicators, and physical and chemical parameters were measured using specified instruments for three days during weekdays. The SBS symptoms were assessed by a questionnaire adopted from the Industry Code of Practice of Indoor Air Quality (ICOP-IAQ) 2010 among 19 employees from the office in East Coast Malaysia. Relationship between past symptoms and present symptoms which are draught (past symptoms) with feeling heavy headed (present symptoms) (*r* = 0.559, *p* < 0.05), room temperature too high (past symptoms) was highly correlated with feeling heavy headed (present symptoms) (*r* = 0.598, *p* < 0.01) and cough (present symptoms) (*r* = 0.596, p < 0.01). Room temperature (past symptoms) has a positive medium relationship with cough (present symptoms) (*r* = 0.477, *p* < 0.05) and scaling itching scalp or ears (present symptoms) has a relationship between stuffy bad air (*r* = 0.475, *p* < 0.05) and dry air (*r* = 0.536, p < 0.05). There was a significant association between RH with drowsiness (χ2 = 7.090, *p* = 0.049) and dizziness (χ2 = 7.090, p = 0.049). The association was found between temperature and SBS symptoms between temperature with headache (χ2 = 7.574, *p* = 0.051), feeling heavy-headed (χ2 = 8.090, *p* = 0.046), and skin rash itchiness (χ2 = 7.451, *p* = 0.044). Air movement also showed a positive association with symptoms of feeling heavy-headed (x2 = 8.726, *p* = 0.021). PM_10_ has positive significance with SBSS which are feeling heavy-headed (χ2 = 7.980, *p* = 0.023), and eyer's irritation (χ2 = 7.419, *p* = 0.038). The conclusion of this study showed that there were positive significant between temperature and relative humidity toward SBSS.

## 1 Introduction

Indoor pollution is mostly caused by inadequate ventilation, a lack of air conditioning systems, human activity, and countless materials, chemicals, and gases [1]. Organizations such as the World Health Organization (WHO) and the United States Environmental Protection Agency (US EPA) have recognized IAQ as a multi-disciplinary phenomenon and classified pollutants into several categories. The WHO estimates indoor air pollution contributed to about 1.5 million deaths in 2000 [2,3]. Additionally, indoor air pollution has been identified as the third most significant contributor to disability-adjusted life years globally which can lead towards Sicks Building Syndrome symptoms [4,5]. Table 1 lists several indoor contaminants and their effects on health which proved that poor IAQ has significance with the SBSS and lead toward decrease productivity of the occupants inside the buildings. The term “sick building syndrome” (SBS) refers to circumstances in which residents of a building feel unwell and uncomfortable, seemingly related to their stay there, even when no particular disease or cause can be found [3]. The complaints could be focused on a specific area or room, or they could affect the entire structure. In addition, the majority of the complainants express relief shortly after departing the premises [6]. The World Health Organization describes SBS as a disorder that affects people who work or live in modern buildings and causes symptoms like weariness, headaches, headaches, and irritation of the skin and mucous membranes which related with poor IAQ ([7]; [8]; [9]). Human health and welfare are significantly influenced by the air quality in interior surroundings [10]. Numerous studies have demonstrated relationships between enhanced interior settings and human health [9]. Low IAQ can result in mortality due to unfavourable health conditions in the worst-case scenarios [11]. Numerous studies have demonstrated relationships between IAQ and SBSS [7,12]. Poor IAQ can result in mortality due to unfavorable health conditions in the worst-case scenarios [13]. This emphasizes how crucial indoor air quality (IAQ) is in any interior setting where people spend most of their time.

**Table 1:** Physical contribution towards SBSS.

Author	Working performances condition	Main conclusion
[14]	380 ± 9 (CO_2_), 27.1 ± 0.1 (AM) 31 ± 3 (RH)	5–10 min-Cannot concentrate and think clearly, fatigue, wellbeing, dizziness, headache. 20–25 min-Dry nose, dry eye, throat, dry skin, unstable mood 30–70 min-Heart rate getting slower
[9]	−21–22^o^ C −23-24 °C −15-30 °C -21^0^c	-Performance's increase -Performance's decrease −10% reduction compared with 21^o^c to 23^0^c
[15]	*T* > 32 °C; RH,60% Hot and humid environment -Poor ventilation	Can caused fainting, muscle cramps heat stroke, exacerbate the underlying medical condition such as heart disease or lung. -Extract bio effluents (odour), reduced productivity rate, nose, rhinitis, throat irritation, headaches, asthma, increased susceptibility to colds or flu and fatigue.
[16]	30 °C	Increase intensity of SBSS Feeling down Lazy Decrease performances, heart rate, respiratory ventilation and end tidal partial pressure of CO_2_ increased due to arterial oxygen saturation decreased
[5]	18.7-25 °C 25.3-28 °C For 8 h working hours	Cognitive tasks of visual reaction time (VRT), subitizing, strop test, backward corsi block tapping (BCBT), N-back and typing Increase performance of occupants-VRT and subitizing increase and BCBT decrease. Only VRT increase significantly
[10]	T > 32 °C *R* > 60%	Muscle cramps, distraction, exacerbation of medical condition
[2]	Summer, winter, and mid-season	The most important factor impacting the degree of satisfaction with thermal comfort was individual ventilation control. More controllability results in higher levels of thermal and visual comfort satisfaction.
[1]	↑RH	Less experience mucosal and cutaneous SBSS
[4]	T too low Varying temperature	Increase fatigue (34.1%), mucosal (19.8%) and dermal symptoms (8.1%)

There were 5 main sectors economy in Malaysia which consists of agriculture, construction, manufacturing, services, mining, and quarrying. Based on Department of Statistics Malaysia (DOSM), 2021 stated that main contributions of employments were services (57.66%), manufacturing (24.07%), construction (12.44%), agriculture (5.19%) and lastly was mining and quarrying (0.64%) in Malaysia. In Terengganu, there were two dominant sectors that increase in 2021 compared to 2019 which are services (3.6%) and manufacturing (1.2%) [17]. Manufacturing in Terengganu was conquered by petroleum and chemical products (96.4%) followed by subsectors transport equipment, others manufacturing and repairs (2.2%) which also involved the largest boat making manufacturing in Peninsular Malaysia in 2021 which showed improvements from previous year. Sectors services in Terengganu consists of subsectors wholesale and retail trade (17.7%), education (5.5%) and hospitality/accommodation (1.2%) which showed positive contribution towards national economy. Increasing both sectors due to the recovery in subsectors such as manufacturing, wholesale and retail trade subsector, hospitality, and education which both sectors were dominants sectors that contribute towards local or global employment. The working environment of both sectors was involved inside the buildings. Good indoor air quality (IAQ) was indirectly important to maintain and increased productivity of the employee in leading the national economy. Numerous short- and long-term health issues can result from poor indoor air quality effect with can triggered to discomfort, absenteeism, ill health in the workplace and cause low productivity of employee [18,19].

Previous studies were summarized in Table 2 which showed variations of air pollutants inside the buildings which can reduced the productivity of the occupants in Malaysia for the past 5 years. Most of the study focused on IAQ parameters while Sick Building Syndrome Symptoms (SBSS) were less conducted in Malaysia besides most of study was conducted in educational zone rather than office area which spend most of their time especially office workers (more than 48%) in same and indoor environments [20]. Therefore, indoor air has a considerable impact on public health, at least as much as outdoor air quality. It is stated in studies conducted on this subject that poor indoor air quality (IAQ) may cause various respiratory diseases, allergic diseases, and cancer. Over the long run, inadequate indoor air quality may lead to illness, absenteeism, loss of concentration, tiredness, drowsiness, and adverse health symptoms such as respiratory problems or headache, and decreased activities performances [21,22]. Services are the sector that employs more people, followed by industry, manufacturing, construction, and agriculture [23]. Nowadays, a lot of service work is done in office buildings, which are frequently distinguished by their sealed facades, increased use of mechanical ventilation and air conditioning, and various electronic equipment like computers, printers, monitors, and audiovisual conference equipment [24]. Since many employees spend a significant portion of their daily lives in shared workspaces, office buildings must provide comfortable interior surroundings for all users [25]. It is commonly known that indoor air quality (IAQ) has a significant impact on occupants' circumstances and can be a critical element influencing health and well-being and determination relationship between IAQ and SSS was crucial to improved productivity of the occupants in workplace [26].

**Table 2:** Previous study between IAQ and SBSS.

Author	Types of Building	SBS Questionnaire	Physical parameter	IAQ monitoring Parameter besides physical parameter					
				CO_2_	HCHO	TVOC	PM_2.5_	PM_10_	CO
[27]	Factory		X	X		X	X	X	X
[7]	Educational	X	X	X	X		X	X	
[28]	Educational			X		X	X		X
[12]	Office	X		X		X	X	X	X
[29]	Educational		X	X				X	
[30]	Educational		X	X			X	X	
[31,32]	Educational		X	X	X	X	X		
[33]	Educational		X	X			X		

There are various reason why indoor air pollutants must be conducted such as unidentified sources of indoor pollutants, potential adverse health effects have been known, occupants and contractors of the buildings want to accomplish the limit values or recommended guidelines towards the pollutants and suitability of monitoring [34]. Improved remediation of air quality issues, particularly with regard to improvement for human adverse health consequences like SBSS, will result from better IAQ data quality and data analytics. Furthermore, field study findings and indoor air quality measurements are valuable sources of information for legislators and regulators who set building rules and standards. If, however, the purpose is to take legislative and regulatory action, the data must be complete and given in a comprehensible and uniform style.

This research aims to determine relationship between IAQ with SBSS by assess IAQ complies with standards for air pollutants and determine significant of SBSS and IAQ, which can prevent discomfort or adverse health impacts for workers. A healthful indoor work environment must have good IAQ [27]. Numerous acute and chronic health issues can be brought on by poor indoor air quality [31,32]. Poor IAQ is frequently linked to respiratory issues, allergic reactions, eye irritation, sinusitis, bronchitis, and pneumonia, among other health issues [31,32,12].

## 2 Methodology

### 2.1 Study area

**Fig. 1:**
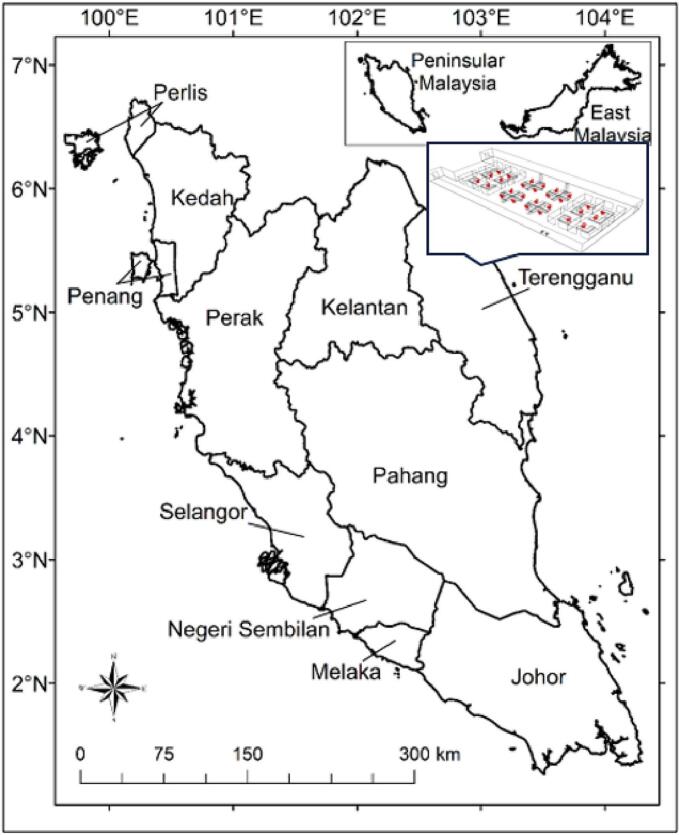
Location of study area

This study was conducted inside one of the administration offices in public university in the East Coast Peninsular Malaysia. This study was conducted inside the office due to administrations and services was highest contribution in East Coast area. There were 19 people or so-called respondent that involved in this study (5° 24′ 34.992″ N, 103° 5′ 22.272″ E). Layout of the study area was displayed in Fig. 1 which consists of location and arrangement of the furniture inside the study area.

### 2.2 Data collection

The data was collected for 3 days with 10 min time interval from 0900 to 1700 h. The sampling was conducted during working day. The parameters measured consists of chemical, physical and ventilation performances indicator. Chemical parameter consists of carbon monoxide (CO, ppm), formaldehyde (HCHO, ppm) and respirable particulate matter (RSP, mg/m^3^). Physical parameters were air movement (AM, m/s), relative humidity (RH, %) and temperature (T, ^0^C). Table 3 showed the list of instruments used during the sampling period. Performance ventilation indicators was carbon dioxide (CO_2_, ppm) also was measured to determine the concentration of the pollutants inside the workplace.

**Table 3:** List of instruments.

Instruments	Parameters
TSI Climomaster Model 9545	Temperature, relative humidity, and air movement
DustTrak DRX Aerosol Monitor 8533	RSP (PM_10_)
Q-Trak Indoor Air Quality Monitor 7575	Carbon dioxide and carbon monoxide
Formaldehyde meter	Formaldehyde

All occupants in the study area were evaluated (*n* = 19). Those who worked less than 3 months were excluded from the study (*n* = 6) which was on internship. Questionnaire was distributed to determine the comfortability of the workers and are there Sicks Building Syndrome Symptoms (SBSS) occurred among the occupants or workers. All the occupants were answered the questionnaire which divided into two sections. Section 1 was called general information; Section 2 was symptoms. Section 1 consists of background factors (gender, age and do you smoke?) and environmental conditions, Section 2 consists of present and past symptoms. The questionnaire was taken from Industrial Code of Practice on Indoor Air Quality (ICOP-IAQ) 2010 by Malaysian Department of Occupational Safety and Health (DOSH).

Table 4 shows the details of each questionnaire. Respondents need to choose only one answer for each question. The purpose of the questionnaire was to help identify possible sources of indoor air quality (IAQ) pollutants and potential adverse health effects linked with pollution exposure [35,33]. The answers to the questionnaire would be kept private. The internal consistency or dependability between several objects, measurements, or ratings is measured by Cronbach's alpha [36]. Stated otherwise, it assesses the degree of reliability found in a subject's evaluation of an instrument or a questionnaire's domain, so revealing the stability of the instruments [13]. Cronbach created alpha, which was first employed to assess a psychometric instrument's dependability. Higher values of Cronbach's alpha, which range from 0.5 to 0.99, suggest that the items measure the same dimension which is significant to proceed the questionnaire [37]. A low Cronbach's alpha value (<0.5), on the other hand, indicates that some or all of the items do not measure the same dimension [38]. This study proceed due to the reliability test (Cronbach Alpha) of each item or questions was more than 0.8 in this questionnaire.

**Table 4:** Details of the questionnaires.

**Section 1**	
**Question**	**Selected Answer**
Gender	Male OR Female
Age	<25 yrs. OR 25–39 yrs. OR 40-55 yrs. OR > 55 yrs
Do you smoke?	Yes OR No
Indicate if your work with or near the equipment	
a) Video display unit computer b) Photocopier c) Fax machine	Everyday OR 2–3 times weekly OR Never


**Section 2**	
**Question**	**Selected Answer**
Past Symptoms	
a) Draught b) Room temperature too high c) Varying room temperature d) Room temperature too low e) Stuffy “bad air” f) Dry air g) Unpleasant odour h) Passive smoking i) Dust and dirt	Yes, often (every week) OR Yes, sometimes OR No, never
Present Symptoms	
a) Headache b) Feeling heavy headed c) Fatigue/ lethargy d) Drowsiness e) Dizziness f) Nausea/vomiting g) Cough h) Irritated, stuffy nose. i) Hoarse, dry throat j) Skin rash/ itchiness k) Irritation of eye l) Scaling/ itching scalp or ears	Yes, often (every week) OR Yes, sometimes OR No, never

### 2.3 Data analysis

Descriptive analyses were used to determine relationship between indoor and SBSS relationship in the study area by using Microsoft Excel and Statistical Package for the Social Sciences (SPSS ®) version 22.0. Data analysis consists of two types which are descriptive analysis and inferential statistics. Descriptive analysis is a fundamental component in helping to analyse the data and is used as brief descriptive coefficients that summarize the data set, which can be either a representation of the entire or a sample of a population [39] and mean used in this study to compare with the standard of IAQ.

Multivariate statistical analysis or inferential statistics was used to specify the analysis. The analysis applies several techniques to determine the sources of pollutants and helps to examine the relationship between air pollutants and meteorological parameters [40,41]. Correlation analysis was used to determine the relationship between qualitative and quantitative data sets and this is important to help in deciding significant data in this study [42]. Chi-square was used to determine the association between IAQ and SBS symptoms and this test is used to explore the relationship between two categorical variables [11,43].

## 3 Result

### 3.1 Trend of all parameters

**Fig. 2:**
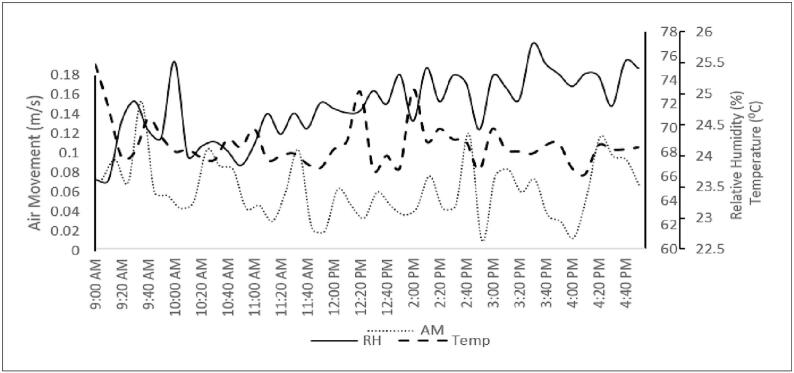
Trend for physical parameters at study area.

**Fig. 3:**
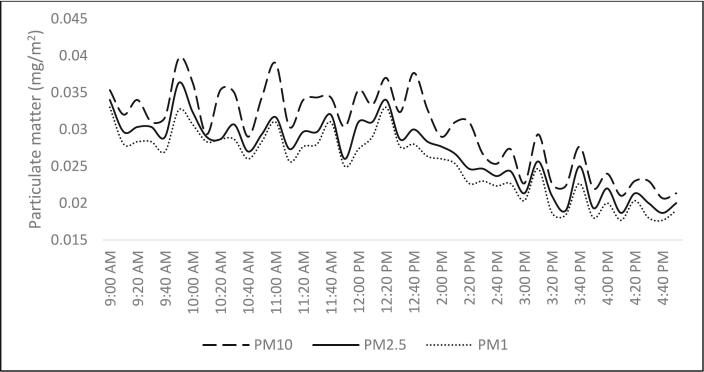
Trends of air pollutants.

**Fig. 4:**
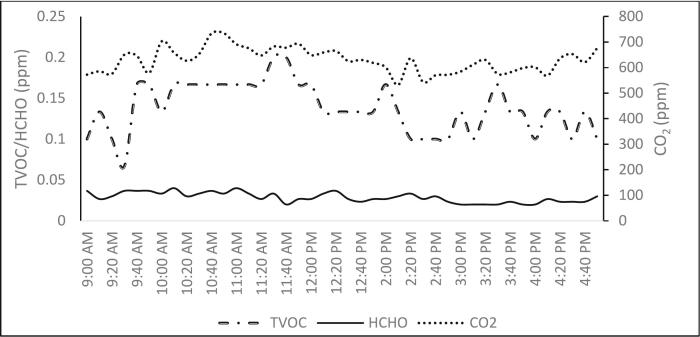
Trends of chemical parameters and ventilation performances indicators.

Trend of physical, chemical and ventilation performances indicator were showed in Fig. 2, Fig. 3, and Fig. 4. Physical parameters were complied with ICOP-IAQ 2010 except for air movement which is too low from the standard. The other parameters complied with ICOP-IAQ 2010. Temperature and relative humidity trend were inversely proportional. Temperature increases lead to a decrease in relative humidity; thus, the air will become drier whereas when temperature decreases, the air become wet means the relative humidity was increase.

### 3.2 Spatial trend of IAQ

**Fig. 5:**
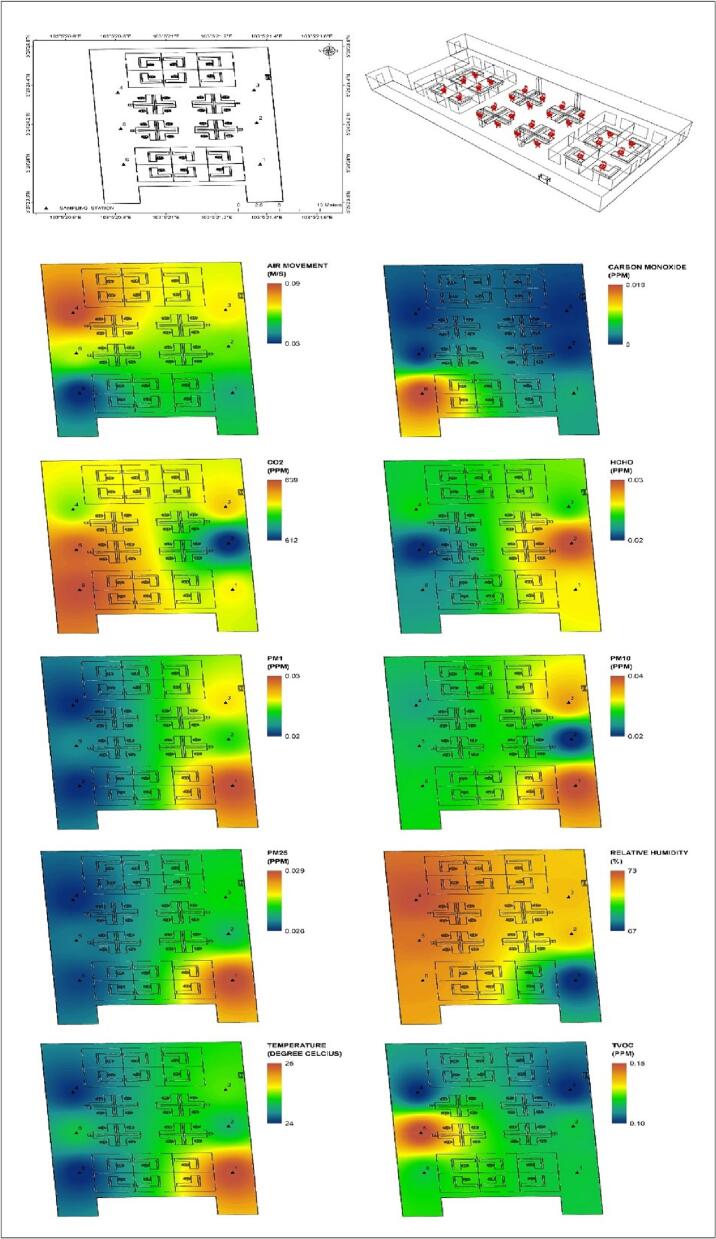
Spatial mapping at study area.

Distribution of the air pollutants was conducted using Geographic Information System (GIS). Spatial mapping inside the office can increase awareness of the occupants to reduce the sources of air pollutants compared to the current situation which widely used for indoor and ambient air. Fig. 5 showed that high air movement can reduced the air pollutants inside the building. Sources of the pollutants need to identify which directly can increase the comfortability of the occupants.

### 3.3 I/O RATIO

I/O ratio, which is very simple to figure out and frequently used, directly illustrates the link between indoor and outdoor particle concentrations. To give a broad overview of the link between indoor and outdoor particles, I/O ratio statistics were thus summarized. Table 5 showed that I/O ratio was in between 1.01 and 1.29.

**Table 5:** I/O ratio.

	I/O Ratio
T (°C)	1.00
AM (m/s)	1.29
RH (%)	1.01
CO_2_	1.01
TVOC	1.03
Formaldehyde	1.04
PM_10_	1.02
PM_2.5_	1.05
PM_1_	1.05

### 3.4 Trend of SBSS

Demographic factors of the respondents were collected which consists of gender, age, and other's opinion. 42.11% (*n* = 8) was male and others 57.89% (*n* = 11) was female. Age of the workers was divided into 3 part which are less than 25 years old, 25 to 39 years old and 40 to 49 years old. Table 1 shows demographic results for the workers at administrative office. The SBSS questions was “When do the symptoms occur?” eithers it is in the morning, evening or unknown. Besides that, the questions also ask about when do the respondents experience relief from these symptoms? And the answers provided was after leaving the building, after leave working place and not sure. The others question was “Do the respondent's smoke?” and indicates if your work with or near the equipment such as video display unit computer, photocopier, and fax machine. Results of this study showed that 57.89% of female has evening symptoms in this workplace and others 42.11% was contribute by male which 10.53% was agreed that the symptoms of Sick Building Syndrome Symptoms (SBSS) in morning, 15.79% in evening and 15.79% was unknown (Table 6). Next questions were “Do you smoke?” and 68.42% of the respondents was not smoking which 57.9% was female and others was male. Others 31.58% was smoking which are 15.79% was frequently smoking and others 15.79% was sometimes smoking. Equipment inside the office was used in the office was female which 52.63%–21.06%. Frequency of video display unit computer was used every day for 73.16% which 10.53% (male) and 52.63% (female). Photocopier was used up to 68.42% and fax machine was used 31.58%. Respondents was divided into a few categories of age ranged, most of respondent was ranged between 25 and 39 years old (63.16%) and aged between 40 and 49 years old (31.58%).

**Table 6:** Results for demographic factors.

		Gender	
		Male (%)	Female (%)
Age	<25 years old	1(5.26)	0
	25–39 years old	6(31.58)	6(31.58)
	40–49 years old	1(5.26)	5(26.32)
When do the symptoms occur?	Morning	2 (10.53)	0
	Evening	3(15.79)	11(57.89)
	Unknown	3(15.79)	0
When do you experience relief from these symptoms?	After leaving the building	0	3(15.79)
	After leave working place	7(36.84)	4(21.05)
	Not sure	1(5.27)	4(21.05)
Do you smoke?	Yes	3(15.79)	0
	No	2(10.52)	11(57.9)
	Sometimes	3(15.79)	0

Indicate if your work with or near the equipment:			
Video display unit computer	Everyday	2(10.53)	10(52.63)
	Never	6(31.58)	1(5.26)
Photocopier	Everyday	3(15.79)	10(52.63)
	Never	5(26.32)	1(5.26)
Fax machine	2–3 times weekly	3(15.79)	3(15.79)
	Everyday	0	1(5.27)
	Never	5(26.32)	7(36.83)

**Fig. 6:**
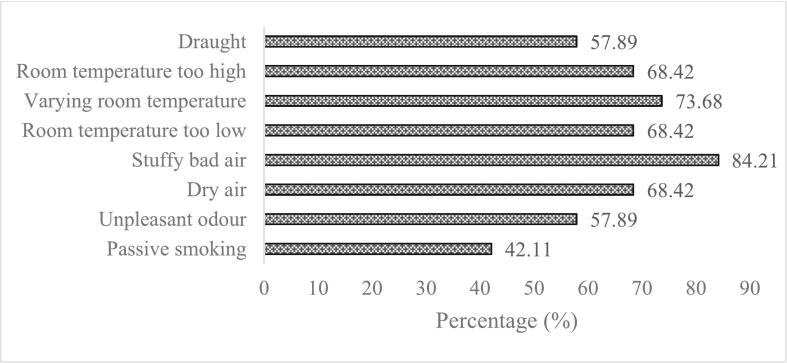
Percentage of the past symptoms in workers.

**Fig. 7:**
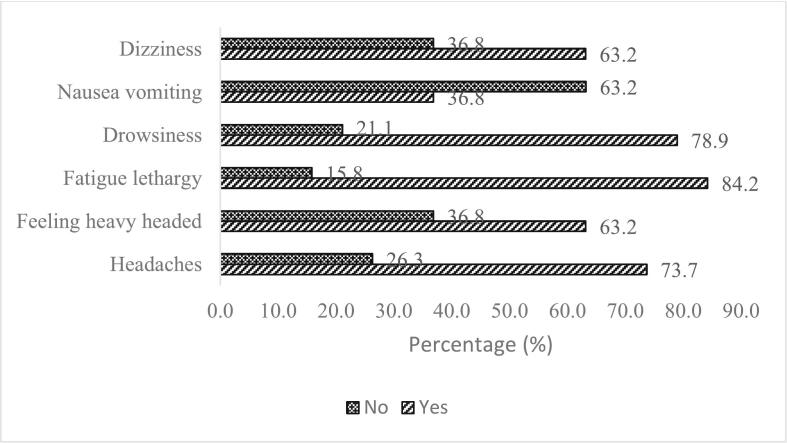
Percentage of the present symptoms in workers.

Questionnaire consists of the symptoms for past 3-month symptoms consists of several symptoms which showed in Fig. 6 and Fig. 7 showed past 3-month symptoms which 84.21% of the respondents agreed that the working place was stuffy bad air and indirectly cause fatigue lethargy (84.21%). The answered consists of “Yes, sometimes” which represents 2 to 3 times week, “Yes, often” which the workers feel the symptoms every day and “No, never” which never feel the symptoms during working hours and the Fig. 2 showed the result of the respondents that is “Yes, often”. Highest condition that experiences by the workers was 84.21% was stuffy “bad air”, this is due to the insufficient air movement inside the building. Second was varying room temperature (73.68%), room temperature too high (68.42%) and dry air (68.42%). These three past symptoms showed insufficient of ventilation inside the building. Fig. 2 showed percentage of the past symptoms in workers which most of the past symptoms more than 50% faced by the workers and this may lead to decrease efficiency and health of the workers for long term. Poor air quality in a building significantly influences work productivity and may contribute to SBS. Many results demonstrate that several personal traits, sociodemographic factors, workplace conditions, and IAQ parameters are related to SBS symptoms. Ventilation rate, chemical pollutants, and physical contamination all have documented links to SBS symptoms. Fig. 3 showed percentage of present symptoms. Overall results showed that present symptoms were faced by the occupants more than 50%. Most of the occupants was feeling fatigue lethargy (84.2%), drowsiness (73.68%), dry air (68.42%), and room temperature too low (68.42%).

### 3.5 Relationship of demographic of IAQ with SBS

After measure trend of IAQ and questionnaire the analysis proceeds with determination relationship between of demographic factors, SBSS (past 3-month symptoms and present symptoms) and IAQ. Results at Table 7 showed strong relationship between the reliefs and the times of the symptoms (*r* = 0.557, *p* < 0.05) occurs was contribute towards drowsiness. There was strong relationship between temperature and demographic factors which are age (*r* = 0.606, *p* < 0.01). Table 4 showed that there was positively high correlation between age and temperature (r = 0.606, p < 0.01).

**Table 7:** Relationship of demographic with SBSS and IAQ.

	Age	When do the symptoms occur?	When do you experience relief from these symptoms
	r	r	r
Drowsiness		0.557⁎	0.750⁎
Temperature	0.606⁎⁎		

⁎⁎ Correlation is significant at the 0.01 level (2-tailed).

⁎ Correlation is significant at the 0.05 level (2-tailed).

Present and past symptoms was evaluated to determine the relationship of the past and present symptoms and Spearman correlation analysis was used. Table 8 showed relationship between past symptoms and present symptoms which are draught (past symptoms) with feeling heavy headed (present symptoms) (*r* = 0.559, *p* < 0.05), room temperature too high (past symptoms) was highly correlated with feeling heavy headed (present symptoms) (*r* = 0.598, *p* < 0.01) and cough (present symptoms) (*r* = 0.596, p < 0.01). Varying room temperature (past symptoms) has positive medium relationship with cough (present symptoms) (*r* = 0.477, p < 0.05) and scaling itching scalp or ears (present symptoms) has relationship between stuffy bad air (*r* = 0.475, p < 0.05) and dry air (*r* = 0.536, p < 0.05). Present symptoms were irritation of eyes has positively correlated with past symptoms which are unpleasant odour (*r* = 0.558, *p* < 0.005), stuffy bad air (*r* = 0.549, *p* < 0.001) dust and dirt (*r* = 0.460, p < 0.005).

**Table 8:** Correlation of present and past symptoms.

		Present Symptoms					
		Feeling heavy headed	Nausea vomiting	Cough	Skin rash itchiness	Irritation of the eye	Scaling itching scalp or ears
Past Symptoms		r	r	r	r	r	r
	Draught	0.559⁎					
	Room temperature too high	0.598⁎⁎		0.596⁎⁎			
	Varying room temperature			0.477⁎			
	Stuffy bad air					0.549⁎	0.475⁎
	Dry air						0.536⁎
	Unpleasant odour				0.495⁎	0.558⁎	
	Passive smoking		0.467⁎				
	Dust and dirt					0.460⁎	

⁎⁎ Correlation is significant at the 0.01 level (2-tailed).

⁎ Correlation is significant at the 0.05 level (2-tailed).

### 3.6 Chi Square of present SBS with IAQ

Chi square analysis IAQ was categorized into high concentration (High) and low concentration (Low) depending on the mean value, either above or below the mean which showed in Table 9. Most of the IAQ parameters did show a significant association with SBS symptoms especially temperature and relative humidity parameters. Association was found between temperature and SBS symptoms which showed positive association between temperature with headache (χ2 = 7.574, *p* = 0.051), feeling heavy headed (χ2 = 8.090, *p* = 0.046) and skin rash itchiness (χ2 = 7.451, *p* = 0.044). PM_10_ has positive significant with SBSS which are feeling heavy headed (χ2 = 7.980, *p* = 0.023), and eyer's irritation (χ2 = 7.419, *p* = 0.038).

**Table 9:** Association of SBS Symptoms.

**Temperature**					
	Yes, often	Yes, sometimes	No, never	X^2^	*p*-value
	Headache				
Low	3 (15.79)	9 (47.37)	4 (21.06)	7.574	0.051*
High	1(5.26)	1 (5.26)	1 (5.26)		
	Feeling heavy headed				
Low	4 (21.06)	6 (31.58)	6 (31.58)	8.090	0.046*
High	1 (5.26)	1 (5.26)	1 (5.26)		
	Skin rash itchiness				
Low	2 (10.53)	4 (21.06)	10 (52.63)	7.451	0.044*
High	1 (5.26)	0	2 (10.53)		
**Relative Humidity**					
	Yes, often	Yes, sometimes	No, never	X^2^	p-value
	Drowsiness				
Low	4 (21.06)	4 (21.06)	2 (10.53)	7.090	0.049*
High	4 (21.06)	3 (15.79)	2 (10.53)		
	Dizziness				
Low	4 (21.06)	5 (26.31)	1 (5.26)	8.041	0.018*
High	0	3 (15.79)	6 (31.58)		
**Air Movement**					
	Yes, often	Yes, sometimes	No, never	X^2^	p-value
	Feeling heavy headed				
Low	2 (10.53)	5 (26.31)	2 (10.53)	8.726	0.021*
High	3 (15.79)	2 (10.53)	5 (26.31)		
**Particulate Matter (PM**_**10**_**)**					
	Yes, often	Yes, sometimes	No, never	X^2^	P-value
	Irritation of the eye				
Low	0	1 (5.26)	1 (5.26)	7.419	0.038*
High	3 (15.79)	7 (36.85)	7 (36.84)		

## 4 Discussion

### 4.1 Trend of all parameters

The optimum interior temperature varies from 23 °C to 26 °C, while the appropriate indoor relative humidity (RH) ranges from 40% to 70%, according to ICOP 2010. The permitted range typically exceeds the ASHRAE Standard 55 recommendations. All-natural ventilated classroom temperatures in Malaysia are between 28 °C and 34.90 °C, which are outside of the body's comfort range due to the country's tropical climate. According to Salleh et al. [44,45] naturally ventilated classrooms had mean inside temperatures that were higher than the outside temperature. From 9.00 am to 12.00 pm, the temperature profile gradually increases. Most air-conditioned inside the office were within the acceptable range of indoor temperatures. Sun et al. [1] reported that all air-conditioned classrooms had indoor relative humidity readings between 44.3% and 47.5%, which were within the acceptable range. Natural ventilation, ranging from 58.48% to 77.5%, however, recorded a mean indoor relative humidity that was greater than advised. Between 7:00 and 8:00 in the morning, there was a larger percentage of indoor relative humidity recorded, and the value decreased with time dependent on temperature [46]. The concentrations of some indoor pollutants can also increase in response to changes in temperature and relative humidity. It's important to keep indoor relative humidity levels within a particular range because low percentages can lead to issues like dry eyes, noses, ears, and throats while high percentages encourage the growth of mold.

By monitoring the CO_2_ level of the indoor environment, one may determine the ventilation rate and indoor air quality. ICOP 2010 recommends that CO_2_ exposure not exceed 1000 ppm for a period of 8 h, with mean and median readings ranging from 537.04 ppm to 1680.35 ppm, ten studies examined indoor CO_2_ levels. Buildings in urban settings produced higher levels of CO_2_ than suburban and rural offices. Some investigations found that during indoor activities such as meeting in the small area, CO_2_ levels especially in office near 1000 ppm. Indoor CO_2_ concentration rose throughout peak hours, fell at break (10 am). This circumstance demonstrated that the main source of indoor CO_2_ was respiratory activity towards occupants. During the break period, CO_2_ concentrations decrease when windows and doors are opened, and occupancy is decreased. The type of ventilation employed and the number of occupants in the room also affect the CO_2_ level inside the building. Furthermore, [40] discovered a strong correlation between occupancy and high CO_2_ levels, which was even more pronounced in the air-conditioned kindergarten. A study was done on five office that use various ventilation methods, with four of ten office utilizing air conditioners and the rest using natural ventilation with one ceiling fan per class and fitted with a fan blade. Between 19 and 21 occupants can fit in each room. CO_2_ values in all four groups were greater than 1000 ppm. The results of this investigation were consistent with those of Razali et al. et al. since al of the air-conditioned interior buildings also recorded CO_2_ levels between 1068 and 1090 ppm with 16, 19, and 24 occupants, respectively. Most structures with natural ventilation recorded CO_2_ levels under 1000 ppm. Nahar and Salleh discovered that each building using natural ventilation had high occupant densities, which also led to high CO_2_ levels. Inadequate ventilation, a lack of windows, and brief window opening times are some of the contributing causes. According to the results, rooms with a 2m^2^/person density seemed to have satisfactory indoor air quality, with indoor CO_2_ mean value concentrations below the ICOP 2010 standards.

Twelve papers reported on the levels of indoor particulate matter inside the buildings. Twelve (12) of those studies reported PM_10_, six (6) reported PM_2.5_, two (2) reported PM_1_, and one (1) reported PM_0.1_. According to ICOP 2010, the acceptable particulate matter limit is 0.15 mg/m^3^, and according to publications we read, most buildings with natural ventilation had high PM_10_ and PM_2.5_ levels. Four out of the twelve studies that captured PM_10_ values discovered that the readings were higher than 100 mg/m^3^ which can be caused by external and internal factors. The results of a study by Salleh et al. [44,45] in five urban buildings showed that the mean concentration of indoor particulate matter in naturally ventilated ranged from 158.57 mg/m^3^ to 316.24 mg/m^3^, while the value of indoor PM_10_ mean concentration ranged from 41.00 mg/m^3^ to 73.48 mg/m3 were within the recommended value.

### 4.2 Spatial trend of IAQ

Previous research suggested that the main sources of indoor HCHO levels were construction materials, furniture, or consumer goods. Additionally, the levels of HCHO indoors were influenced by temperature, which was higher in tropical and subtropical regions than in other climate zones. Therefore, it was determined in this study that indoor temperature and the sources of pollution were significant factors affecting indoor pollutants. Previous research has shown that cooking, inadequate ventilation rates, and population density all contribute to elevated indoor CO_2_ levels. The eastern Taiwanese residences under study had inferior ventilation rates. The effects of these elements should therefore be evaluated in the future to fully comprehend the high CO2 levels in eastern Taiwan. According to the publications we read, most buildings with had high PM_10_ and PM_2.5_ levels. Four out of the twelve studies that captured PM_10_ values discovered that the readings were higher than 100 g m-3. In five metropolitan kindergartens with 10 classrooms, a study by Salleh et al. [44,45] revealed that the mean concentration of indoor particle in classrooms with natural ventilation ranged from 158.57 mg/m^3^ to 316.24 g/m^3^. On the other hand, indoor PM_10_ mean concentration values in air-conditioned classrooms ranged from 41.00 g/m^3^ to 73.48 mg/m^3^, which were within the acceptable range. According to Salleh et al. [44,45] the kindergarten's location, which was between 20 and 500 m from the major road and surrounded by a commercial area, was a factor in the higher interior particulate matter levels.

The term “indoor air pollution” (IAP) refers to the contaminants that are present in interior settings, and it has recently grown significantly because of the lengthened time that people spend indoors. IAP exposure has been linked to serious health issues like cancer, sick building syndrome, and respiratory and cardiovascular conditions. Indoor pollution sources that emit gasses and particles into the atmosphere are the main causes of indoor air quality (IAQ) issues. Inadequate ventilation prevents emissions from these sources from being sufficiently diluted, which is another prevalent cause of air quality problems. In this blog post, we outline some major pollutants' health effects and their origins, as well as some actions you may take to enhance the quality of the air within your home. The impact of other contributing parameters, such as penetration factor, deposition rate, indoor particle source emission rate, and outdoor particle concentration, depends on the air exchange rate inside the building. For instance, if there is no indoor particle source, air movement will result in a reduction in pollutant concentration. The concentration of pollutants will, however, fall with an increase in air exchange rate if the indoor particle source emission rate is very high and the outdoor particle concentration is very low.

### 4.3 I/O RATIO

The link between indoor and outdoor concentration was clearly expressed by the I/O ratio, which is extensively used and relatively simple to understand. To give a broad overview of the link between indoor and outdoor particles, I/O ratio statistics were presented. To assess the variation between indoor concentration and comparable outdoor levels, the indoor-outdoor ratio is used [47]. I/O ratios greater than or equal to 1.2 show that the indoor concentration is higher than the outdoor concentration and could be a result of indoor sources, 0.8–1.2 or greater show that the indoor concentration is equal to the outdoor concentration, and I/O less than or equal to 0.8 show that the indoor concentration is lower than the outdoor concentration, showing the potential for outdoor influence. The amount of outside pollution, the amount of pollution transported indoors, the presence of indoor sources, and the presence of RSP are all factors that affect its presence indoors [48].

The penetration factor, air exchange rate, and deposition rate can all have an impact on the infiltration factor. The air exchange rate, particle size, indoor/outdoor pressure difference, penetration factor, and geometry of building envelope cracks all play a role in infiltration ventilation of a structure. Wind direction and speed are influencing elements as well because they have an impact on the air exchange rate and pressure difference between indoors and outdoors. Again, since it is challenging to quantify the specific information on these contributing elements, most of this research are unable to do so. Additionally, the infiltration factor can be significantly impacted using mechanical ventilation with an air filter. The infiltration factor can be decreased with higher filter efficiency.

The penetration factor, air exchange rate, and deposition rate can all influence the infiltration factor. The air exchange rate, particle size, indoor/outdoor pressure difference, penetration factor, and geometry of building envelope cracks all play a role in infiltration ventilation of a structure. Wind direction and speed are influencing elements as well because they have an impact on the air exchange rate and pressure difference between indoors and outdoors. Again, since it is challenging to quantify the specific information on these contributing elements, most of this research are unable to do so. Additionally, the infiltration factor can be significantly impacted using mechanical ventilation with an air filter. The infiltration factor can be decreased with higher filter efficiency. Through door monitoring, the influence of occupant behaviour on changing I/O ratios can be more specifically studied and can caused indoor concentration is equal to the outdoor concentration. Previous study the 18 units underwent measurements as well as recordings of window use (open/closed) and occupancy can caused [49]. This enables a more in-depth analysis of how occupant behaviour affects the I/O ratio and indoor air quality in general.

### 4.4 Trend of SBSS

Basically, the physical parameters were too low and exceed the limit especially for temperature and relative humidity showed that the building was inconvenient for occupants [12]. Standard value of the temperature was 23 to 26^0^c while the study area measurement was 22 to 29.9^0^c. For instance, during the winter, countries with moderate or cold climates have been found to be more at risk for cardiovascular and respiratory morbidity and death when interior temperatures are below 18 °C. [50]. Temperatures above 26 °C, on the other hand, might exacerbate acute symptoms including weariness, depression, and poor focus. When temperatures rise above 30 °C, respiratory health may also be significantly affected. High humidity levels can also increase job performance and lessen health issues like dry eye symptoms compared to the circumstances found in offices with excessively dry air. [50]. Temperature and relative humidity are different, but they are related to each other. The formula describing the relationship between temperature and humidity simply states that they are inversely proportional. When the temperature rises, the relative humidity will fall, making the air drier; when the temperature falls, the air will get wet, making the relative humidity rise. Air movement was lower than 0.15 m/s due to the adjusting of air conditioning by the workers or occupants. This study was proceeded with correlation analysis of sick building syndrome symptoms (SBSS) from 3 month before and current symptoms. This is important to determine either there was relationship between the air quality and the symptoms that faced by the occupants or so-called workers in the study area.

The risk of SBS was discovered to be 2.9 times greater in women, 2.8 times higher in those who reported a dusty workplace, and 2.6 times higher in those who reported stuffy “bad” air, dry air, and an unpleasant odour [42]. The SBS may have significant effects on productivity in the workplace. However, given that we spend most of our time at work, extra consideration should be given to how the workplace environment affects SBSS [51]. A few office environment studies with respect to SBSS have been recently performed in Malaysia [40,12], but more investigations are needed since there is a big difference in office environments and SBSS among different areas in Malaysia. Frequency of equipment used such as video display unit computer, photocopier and fax machine were asked towards respondents. Female staff was frequently used by female due to most of administration jobs was conducted by female and for male, their task currently need physical activities such as office boy which assists in menial office tasks required by the office staff besides essential for the efficient operation of a business and offers office support to either a person or a team.

### 4.5 Relationship of IAQ with SBS

Spearman correlation analysis was important to determine the relationship of the possible factors that contribute towards inefficiency of the workers or occupants in the administrative office. Uncomfortable temperature, humidity, physical condition, and psychosocial status are some of the factors identified as root causes of SBS due to the immunity of the occupants itself [52]. Suitable ranged can give comfortability towards occupants but from Table 2 we can see that the temperature was sparked up to 29.9 °C which can give influences towards the occupants especially from the aspect working productivity. Next, the occurrences symptoms were drowsiness which related with the time played importance significance towards occupants. The occurrences were supported by the occupants agree that occurrences of SBSS in the evening up to 73.68%, 10.53% in the morning and the balanced occurrences was unknown. Evening work is seen as a significant source of occupational stress and can have detrimental impacts on one's health, including depression, diabetes, hypertension, and cardiovascular disease. [53]. It is also a significant contributor to human mistake, which in turn results in workplace accidents and injuries. Due to the exhausted besides sleepy after taking an heavy lunch, inconvenience working place and also poor indoor air quality [54]. Drowsiness was relief after leaving the building or workplace was related with poor ventilation inside the buildings. These showed that there were inconvenient situations that faced by the occupants or workers insides the building or workplaces that contributes towards SBSS. There has been evidence to show that there is an association between occurrences of the SBSS at workplace with temperature which good IAQ can improved efficiency of the workers inside the building. Precious study stated that out of 30 studies, on sleep disorders, cancer, metabolic endocrine diseases, reproduction, cardiovascular disease, gastrointestinal disorders, shift work, and health outcomes totaled 14, including 2 studies on sleep disorders. Shift work significantly increased the risk of disorders like breast cancer, diabetes mellitus, preterm delivery, abortion, low birth weight, small-for-gestational-age infants, menstrual irregularities, infertility, ischemic heart disease, and ischemic stroke, according to meta-analyses based on quantitative combination of the data from these studies. Additionally, significantly higher risks of sleep disturbance, prostate cancer, body weight change, metabolic syndrome, infertility, and gastrointestinal issues were also noted in some earlier research.

Higher ambient temperature transiently increased the risk of feeling heavy headed, cough, and scaling itching scalp or ears which requiring emergency department evaluation, and this is in line with previous study with approximate 7.5% higher risk for each 5 °C increment in temperature [10]. This study showed that high temperature caused draught and high room temperature. Strong correlation between headache and outside temperature, irrespective of discharge diagnosis. The only previous large case crossover research of migraines, which only looked at a single temperature cut point of 19.6 °C, failed to detect such a connection. [55]. However, even though higher temperatures are linked to reduce blood pressure, some studies have revealed typically higher prevalence of migraine in warmer seasons29–31. [56]. Further research is necessary to determine whether a higher temperature is also linked to a higher risk of inducing the more frequent headaches that do not necessitate ER evaluation. Systolic and diastolic blood pressure were substantially greater when measured at 15 °C than at 25 °C, with mean differences of 32.2 +/− 4.2, p 0.001, and 19.5 +/− 3.0, p 0.001 for each. The difference in the pulse rate at 15 °C and 25 °C was significant (mean difference 11.1 +/− 3.2, p 0.002).

Exposure to indoor air pollutants can result in asthma symptoms or cause asthma exacerbations and cough [57]. It has been indicated that acute exposure to combustion smoke can induce bronchial irritation, inflammation, and enhance bronchial reactivity, which is regarded as the main mechanism responsible for asthma exacerbation, especially in sensitive group. Unpleasant environment inside the building can caused stuffy bad air, dry air and activities inside the building can caused dust and dirt. Stuffy bad air has highly positive correlation with irritation of eye and scaling itching scalp or ears. Dry air can reduce moisture inside the building and this study in line with the output due to the occupant has positive correlation between dry air and scalping itching scalp or ears [10]. It has been demonstrated that children between 5 and 14 years living in houses combusting coal, wood, and kerosene have a relative risk of 1.6 in asthma exacerbation which include passive smoking can caused vomit/nausea [57].

In the current study, hoarseness/dry throat, a heavy feeling in the head, and trouble concentrating were the most reported symptoms with a perceived causality to the classroom setting, with a prevalence of roughly 70–76%. [58]. With a prevalence of between 35 and 46%, dry/red hands, nausea, and itchy/scaly ears or scalp were the least common office-related complaints. Comparatively speaking, the most common reported symptoms were respiratory, general, and primary headache symptoms, with prevalence of 58.3, 45.3, and 47%, respectively. This is in line with the results of this study which are temperature inside the classroom can caused many symptoms towards occupants such as feeling heavy headed, cough and stuffy bad air can cause irritation of eye and scaling itching scalp or ears. This proved that convenient temperature and air movement can reduced symptoms inside the building. Your eyes may be feeling dry and irritated from air pollution and diminished air quality due to weather. Although eyes are built to naturally shield themselves from outside particles like dust, wind, and very intense light, they must remain open for the purpose of vision. Chronic inhalation of harmful pollutants harms the eyes, causing everything from moderate discomfort to retinal haemorrhage. [51].

### 4.6 Chi Square of present SBS with IAQ

A heat illness is one caused by high temperatures and humidity. You may get an illness while exercising or working in high heat and humidity either inside the building or at ambient air. If your body is overheating, due too high temperature, bumps on your skin which can lead to skin rash, muscle spasms, headache and feeling heavy headed you may have one of the most common heat-related illnesses which are heat rash, heat stroke and heat cramps [10]. Low or high relative humidity also can cause various health problems. The amount of water vapor in the air is known as humidity. Our bodies typically use air to expel the sweat that collects on the skin when we perspire [59]. The body can cool down as a result. When the air is humid, the warm moisture adheres to our skin for a longer period, increasing how hot we feel. [42]. The human body can suffer from several negative impacts from high humidity. It may aggravate sensations of drowsiness and fatigue. Additionally, hyperthermia can result from high humidity. Which lead towards dehydration, fatigue, dizziness, fainting and drowsiness [60]. A significant association was found between RH with drowsiness in this study which in line with previous study. This showed that relative humidity in the study area was too high and can be improved to avoid health problems among the workers or occupants.

Building ventilation patterns are the consequence of a combination of human activity, natural factors, and mechanical ventilation. These forces produce pressure differentials that transport airborne pollutants via any cracks or gaps from places of comparatively higher pressure to areas of relatively lower pressure. Low airflow or air movement caused the pollutants inside the building stagnant and give negative impact towards occupants. Air flow between areas and between a building's interior and outside are significantly influenced by natural factors. Particularly if the building envelope is leaky, the stack effect and wind can overwhelm a structure's mechanical system and impair airflow and ventilation. Pressure of the low air movement can cause discomfort towards occupants which can lead towards serious illness in future.

Air pollution affects how people live their daily lives and even puts human survival in danger. The impact of outdoor air pollution on health is well-known. Numerous health issues and diseases, including cancer, cardiovascular disorders, pulmonary issues, ophthalmic conditions, neurological conditions, and respiratory problems, are brought on by air pollution [61]. Due to multiple innervations on the ocular surface, the cornea is the most sensitive structure in the human body and is therefore particularly susceptible to environmental factors [62]. Because there is just a thin precorneal tear film to protect the eyes from potentially dangerous external material, human eyes are vulnerable to the negative effects of air pollution ([63]; [64]). Inflammation and irritability are the main negative effects of air pollutants including CO and PM on human eyes, with conjunctivitis being a common issue. To understand the effects of environmental contaminants on the ocular surface, numerous research have been conducted. Travelers visiting extremely polluted places where the PM level was five times greater than the WHO annual average limit of 60 g/m3 experienced substantial subclinical ocular surface alterations, according to Saxena and colleagues [65]. According to Versura and colleagues, the combination of air pollutants caused cytological alterations and inflammation in the ocular surface, which added to eye discomfort. [66]. According to a growing number of studies, exposure to cigarette smoke can lead to cataracts, and air pollutants like PM_10_ are linked to glaucoma and allergic conjunctivitis [67,68]. Additionally, exposure to air pollution brought on by traffic is linked to age-related macular degeneration (AMD) [69].

## 5 Conclusion

The results showed that, relative humidity and temperature was played an important role in give comfortable towards the occupants inside the building. Temperature, air movement and relative humidity levels in the administrative office were not within the acceptable limit set by the Malaysian Standard [70]. Additionally, 82.1% of people reported having Sick Building Syndrome (SBS) symptoms. The workers were overwhelmingly affected by fatigue, feeling foggy-headed, and headaches (84.2%, 73.7%, and 63.1%, respectively). This indicated that workers were at risk of suffering SBS related to poor IAQ. Relative Humidity (RH) level had a significant association with drowsiness and dizziness while PM_10_ level had a positive association with feeling heavy headed, and irritation of the eye. Temperature has significant with headache, feeling heavy headed, skin rash and itchiness which reduced productivity of the occupants inside the study area.

## 6 Recommendation

Identifying and quantifying the emission sources and the correlation of each chemical and physical can be added such as by using Principal Component Analysis (PCA) which each contribution can use an orthogonal or also known as uncorrelated transformation to alter the set of observation which possibly correlated variables into a set of values and linearly uncorrelated variables called principal components (PCs). Improvement and further studies can help authorities in identifying and quantifying the indoor air pollutants sources besides its contributors which are urgently needed for indoor air quality management and advanced modelling of air quality. Further research in IAQ data is also beneficial as it is more inclined to affecting human health and the environment when the data is ready as it has been highlighted Industry Code of Practice of Indoor Air Quality (ICOP-IAQ) 2010 [70].

## CRediT authorship contribution statement

**Amalina Abu Mansor:** Writing – original draft, Investigation, Formal analysis, Data curation. **Samsuri Abdullah:** Writing – review & editing, Validation, Supervision, Conceptualization. **Aimi Nursyahirah Ahmad:** Writing – original draft, Visualization, Formal analysis, Data curation. **Ali Najah Ahmed:** Writing – review & editing, Supervision. **Mohammad Fakhratul Ridwan Zulkifli:** Investigation, Conceptualization. **Suriani Mat Jusoh:** Resources, Conceptualization. **Marzuki Ismail:** Writing – review & editing, Validation, Supervision, Conceptualization.

## Declaration of competing interest

The authors declare the following financial interests/personal relationships which may be considered as potential competing interests:

Samsuri Abdullah reports financial support was provided by Malaysia Ministry of Higher Education. If there are other authors, they declare that they have no known competing financial interests or personal relationships that could have appeared to influence the work reported in this paper.
